# Exosomes from patients with septic shock convey miRNAs related to inflammation and cell cycle regulation: new signaling pathways in sepsis?

**DOI:** 10.1186/s13054-018-2003-3

**Published:** 2018-03-15

**Authors:** Juliana Monte Real, Ludmila Rodrigues Pinto Ferreira, Gustavo Henrique Esteves, Fernanda Christtanini Koyama, Marcos Vinícius Salles Dias, João Evangelista Bezerra-Neto, Edécio Cunha-Neto, Flavia Ribeiro Machado, Reinaldo Salomão, Luciano Cesar Pontes Azevedo

**Affiliations:** 1 Research and Education Institute, Hospital Sirio-Libanes, Rua Professor Daher Cutait 69, São Paulo, SP 01539-001 Brazil; 2 Sao Paulo State Cancer Institute, University of São Paulo, São Paulo, Brazil; 3 Hospital do Servidor Publico Estadual de São Paulo, São Paulo, Brazil; 4 Morphology Department, Instituto de Ciencias Biologicas, Universidade Federal de Minas Gerais, Belo Horizonte, Brazil; 5 Laboratory of Immunology, Heart Institute, University of São Paulo, School of Medicine, São Paulo, Brazil; 6 Universidade Estadual da Paraíba, Centro de Ciências e Tecnologia, Campina Grande, Brazil; 7 Ludwig Institute for Cancer Research, São Paulo, Brazil; 8 International Research Center, A.C.Camargo Cancer Center, São Paulo, Brazil; 9 Division of Clinical Immunology and Allergy, School of Medicine, University of São Paulo, São Paulo, Brazil; 10 Federal University of São Paulo, São Paulo, Brazil; 11 Emergency Medicine, University of São Paulo, São Paulo, Brazil

**Keywords:** Sepsis, Extracellular vesicles, Exosomes, MicroRNAs, Messenger RNA, Inflammatory response, Oxidative stress

## Abstract

**Background:**

Exosomes isolated from plasma of patients with sepsis may induce vascular apoptosis and myocardial dysfunction by mechanisms related to inflammation and oxidative stress. Despite previous studies demonstrating that these vesicles contain genetic material related to cellular communication, their molecular cargo during sepsis is relatively unknown. In this study, we evaluated the presence of microRNAs (miRNAs) and messenger RNAs (mRNAs) related to inflammatory response and redox metabolism in exosomes of patients with septic shock.

**Methods:**

Blood samples were collected from 24 patients with septic shock at ICU admission and after 7 days of treatment. Twelve healthy volunteers were used as control subjects. Exosomes were isolated by ultracentrifugation, and their miRNA and mRNA content was evaluated by qRT-PCR array.

**Results:**

As compared with healthy volunteers, exosomes from patients with sepsis had significant changes in 65 exosomal miRNAs. Twenty-eight miRNAs were differentially expressed, both at enrollment and after 7 days, with similar kinetics (18 miRNAs upregulated and 10 downregulated). At enrollment, 35 differentially expressed miRNAs clustered patients with sepsis according to survival. The pathways enriched by the miRNAs of patients with sepsis compared with control subjects were related mostly to inflammatory response. The comparison of miRNAs from patients with sepsis according to hospital survival demonstrated pathways related mostly to cell cycle regulation. At enrollment, sepsis was associated with significant increases in the expression of mRNAs related to redox metabolism (myeloperoxidase, 64-fold; PRDX3, 2.6-fold; SOD2, 2.2-fold) and redox-responsive genes (*FOXM1*, 21-fold; *SELS*, 16-fold; *GLRX2*, 3.4-fold). The expression of myeloperoxidase mRNA remained elevated after 7 days (65-fold).

**Conclusions:**

Exosomes from patients with septic shock convey miRNAs and mRNAs related to pathogenic pathways, including inflammatory response, oxidative stress, and cell cycle regulation. Exosomes may represent a novel mechanism for intercellular communication during sepsis.

**Electronic supplementary material:**

The online version of this article (10.1186/s13054-018-2003-3) contains supplementary material, which is available to authorized users.

## Background

Sepsis is one of the leading causes of death in intensive care units (ICUs) worldwide. It represents a difficult challenge to the intensivist because this condition lacks good diagnostic tools and adequate evidence-based treatments. The incidence of sepsis has increased considerably in recent years, and the morbidity and mortality rates associated with the syndrome are extremely high, especially for septic shock, in which mortality ranges from 40% to 60% in different populations [1–4].

The molecular basis underlying sepsis mechanisms are complex and still only partially understood [5]. Studies have demonstrated that the pathophysiology of sepsis is related to a dysregulated inflammatory response and oxidative stress [6]. Recently, the presence of circulating extracellular vesicles (EV) shed from different cell types during sepsis and their relationship with sepsis pathophysiology was established [7–9]. Previous studies demonstrated that a special type of EV, named *exosomes* and mostly originated from platelets, induce superoxide production and apoptosis in vascular cells during sepsis by inflammatory and redox mechanisms [10, 11]. Also, our group established a correlation between exosomes and organ dysfunction by demonstrating exosome-induced myocardial depression in isolated heart and papillary muscle preparations. This dysfunction was mediated by a mechanism associated with myocardial nitric oxide production [12].

Recent studies demonstrated that exosomes may play a role in cell-to-cell communication in normal physiology and several diseases, including cancer, through carriage of functional messenger RNA (mRNA) and microRNAs (miRNAs, miRs) [13–15]. Even though the presence of miRNAs in plasma during sepsis was previously demonstrated [16, 17], the genetic content of exosomes during sepsis was evaluated in very few studies [18–20].

In the present pilot study, we isolated exosomes from plasma of patients with septic shock and analyzed their entire miRNA content. We also assessed mRNAs, specifically looking for those related to inflammatory response and oxidative stress, because previous studies by our group and others demonstrated that these mechanisms mediate the effect of exosomes during sepsis [10, 11]. In addition, we correlated the differential expression of miRNA to outcomes and used pathway enrichment analysis to elucidate their most important possible biological mechanisms. miRNA profiling revealed that the genetic material contained in exosomes from patients with sepsis is related to modulation of immune system and cell cycle regulation. The data presented in this article may help to generate new insights into sepsis pathophysiology and ultimately provide research windows to development of novel therapies.

## Methods

A detailed description of the methods is provided in Additional file 1.

### Patients and control subjects

In this prospective observational study, we recruited 24 patients in the initial 24 h of a first episode of septic shock who were admitted to the ICUs of Hospital Sirio-Libanes, Hospital São Paulo/Federal University of São Paulo, and Hospital das Clinicas/University of São Paulo, all in São Paulo, Brazil, from March 2012 to August 2013. Septic shock was defined as the need for vasopressor support after adequate fluid resuscitation [21]. We excluded patients with severe anemia (hemoglobin < 7.0 g/dl) or active bleeding, those with known platelet disorders or conditions that cause thrombocytopenia other than sepsis (e.g., heparin-induced thrombocytopenia or chemotherapy), those with full heparin use (low molecular weight heparin or unfractionated) or use of any other medications that interfere with platelet function, those with a life expectancy no longer than 24 h, and patients with active cancer. The included patients had 30-ml blood samples collected from a central venous or arterial catheter up to 24 h after ICU admission (D0) and from a central venous or arterial catheter or by peripheral puncture 7 days after treatment (D7) for those who survived or were not discharged from the hospital. Twelve healthy volunteers without comorbidities and paired in a 2:1 ratio for sex and age were used as control subjects. The study was approved by the institutional ethics and review board of Hospital Sirio-Libanes (Comitê de Ética em Pesquisa, protocol number HSL 2010/61), which served as the coordinating institution, and also from the other institutions (Comitê de Análise para Projetos de Pesquisa, Hospital das Clinicas, Universidade de São Paulo, and Comitê de Ética em Pesquisa, Universidade Federal de São Paulo). Informed consent was obtained from the patients or their representatives.

### Isolation and characterization of exosomes

Plasma was filtered through 0.2-μm membranes; exosomes were isolated by ultracentrifugation as previously described [12]; and protein extracts were assessed by Western blotting using antibody against exosomal flotillin-1 (ab41927; Abcam, Cambridge, UK). For validation, the number of particles and the particle size were measured using a nanoparticle-tracking analysis device (NanoSight LM10; Malvern Panalytical, Malvern, UK) coupled to a charge-coupled device camera and a laser emitting a 60-mW beam at 405 nm. Video acquisitions were performed in five recordings of 60 seconds each. At least 1000 particles were tracked in each sample. Nano flow cytometry was carried out using human antibodies against cell surface antigens CD9 (exosome marker) and CD41 (platelet marker) using a CytoFLEX flow cytometer (Beckman Coulter Life Sciences, Indianapolis, IN, USA). We used violet side scatter and fluorescent polystyrene beads (Megamix-Plus FSC and SSC; BioCytex, Marseille, France) with known sizes (100, 160, 200, 240, 300, 500, and 900 nm) to identify vesicles smaller than 1 μm. The analysis was performed using CytExpert 2.1 software (Beckman Coulter Life Sciences). Plasmatic concentrations of interleukin (IL)-1β, IL-6, IL-8, IL-10, IL-13, tumor necrosis factor-α, and transforming growth factor-β were measured in seven patients with sepsis by enzyme-linked immunosorbent assay.

### miRNA profiling and inflammatory and oxidative stress gene expression analysis

RNA and miRNA were extracted using the miRNeasy Mini Kit (Qiagen, Valencia, CA, USA) and were reverse-transcribed into complementary DNA (cDNA). Using TaqMan® Array Microfluidic Cards technology (Applied Biosystems/Thermo Fisher Scientific, Foster City, CA, USA) and qRT-PCR, we simultaneously evaluated 754 mature miRNAs (TaqMan MicroRNA Array v3.0; Life Technologies/Thermo Fisher Scientific, Grand Island, NY, USA) and 90 genes related to immune response (TaqMan Low Density Array Immune Profiling; Life Technologies/Thermo Fisher Scientific). The oxidative stress study consisted of 84 genes associated with oxidative stress and antioxidant defense using a commercial PCR array (PAHS065-A; Qiagen). Raw data were analyzed using ExpressionSuite v1.0.3 software (Life Technologies/Thermo Fisher Scientific), and all cycle threshold (*C*_t_) values were evaluated using StatMiner version 5 (Integromics, Granada, Spain). Considering the cDNA preamplification step, *C*_t_ values greater than or equal to 32 for miRNAs and oxidative stress and greater than or equal to 35 for the immune study were excluded according to the manufacturers’ instructions.

For the miRNA analysis, the geNorm method was used to identify the best reference controls. A median of miR-17, miR-20a, and miR-106a expression was then used for data normalization. The expression levels of immune and stress oxidative genes were normalized to 18S ribosomal RNA and β-actin, respectively [22]. The relative gene expression data were calculated using the comparative cycle threshold (2^−ΔΔ*C*t^) method [23].

### Target prediction, network pathway analysis, and cluster analysis

Ingenuity Pathway Analysis (IPA) software (www.ingenuity.com; Qiagen Bioinformatics, Redwood City, CA, USA) and the MicroRNA Target Filter software tool (Qiagen Bioinformatics), which relies on three algorithms (TargetScan, TarBase, and miRecords), were used to identify putative targets of the miRNAs differentially expressed in septic exosomes versus healthy controls and in the comparison of patients with sepsis according to survival. IPA Network maintains a graphical database of networks of interacting genes (Ingenuity Knowledge Base; Qiagen Bioinformatics). Only experimentally validated targets were selected, indicating that only networks already demonstrated in previous studies to be associated with the specific miRNA expression are shown. A list containing the differentially miRNAs was uploaded into the IPA software and analyzed on the basis of content as of October 2016. The significance of the association between each list and the canonical pathways was measured by Fisher’s exact test. As a result, a *p* value was obtained, and values below 0.05 were considered significant. To build the figures, molecules were represented as nodes, and the biological relationship between two nodes was represented as a line. These connections were supported by at least one reference from the literature, from a textbook, or from canonical information stored in the database [24].

Using the normalized 2^−ΔΔ*C*t^ values, we constructed heat maps for comparison of patients with sepsis with healthy control subjects and patients with sepsis according to outcome, where the colors represent a scale of the smaller (blue) to larger (red) expression ratios for each miRNA as compared with the control samples (value = 1/white color). A further analysis by hierarchical cluster based on Euclidean correlation was then done. Each row represents a miRNA, and each column represents a patient sample. The color scale shown in the cluster grouping illustrates the normalized 2^−ΔΔ*C*t^ values for the miRNAs for all samples, where red and blue represent the expression above or below the average, respectively.

### Statistical methods

The statistical tests were applied only to targets expressed in at least 50% of the samples from each group. The 2^−ΔΔ*C*t^ values were tested for normal distribution using the Shapiro-Wilk test. For both paired and nonpaired comparisons, the Wilcoxon test was used. Benjamini-Hochberg adjustment was used to correct *p* values (*p* < 0.05) for false discovery rates [24]. All tests were performed using R software version 3.1.2 (R Foundation for Statistical Computing, Vienna, Austria).

## Results

### Patients’ characteristics and outcomes

Of the 24 patients with septic shock, 18 had blood samples collected both at enrollment and 7 days after. Table 1 shows the main characteristics of the patients. Our patients were relatively younger and with few comorbidities. The organ support at study enrollment was substantial, since 67% of them were mechanically ventilated and all patients were receiving norepinephrine. All but two patients received prophylactic heparin (unfractionated or low molecular weight) during the study period. Red blood cell transfusion was administered to six patients after 2 (1-5) days of inclusion. Two patients received fresh frozen plasma and one patient received platelet transfusion on day one before collection of study samples. The antibiotics most used were ceftriaxone (11 patients), vancomycin (9 patients), meropenem (7 patients), metronidazole (4 patients), piperacillin-tazobactam (4 patients). Other four different antibiotics were also used. Hospital mortality rate was 35%. Healthy volunteers had mean age of 49.7 ± 9.6 years and were predominantly male (58%). None of them reported previous use of medications.

**Table 1:** Demographic data of the included patients

Characteristics	All patients (*N* = 24)
Male sex, *n* (%)	14 (61)
Age, years	53 ± 17
Comorbidities, *n* (%)	
Arterial hypertension	3 (13)
*Diabetes mellitus*	5 (21)
Tobacco use	2 (10)
Others	3 (13)
Origin of patients, *n* (%)	
Emergency room	12 (50)
Ward	5 (21)
Operating room	7 (29)
Source of sepsis, *n* (%)	
Lung	7 (28)
Urinary	5 (21)
Abdominal	5 (21)
Other sources	5 (21)
Undetermined	2 (8)
Hemodynamic data	
Norepinephrine dosage D0, μg/kg/minute	0.3 (0.1–0.5)
Lactate D0, mg/dl	22 (18–36)
Mean arterial pressure D0, mmHg	70 (63–78)
Ventilatory data	
Use of mechanical ventilation D0, *n* (%)	16 (67)
Tidal volume D0, ml	500 ± 88
PEEP D0, cmH_2_O	8 ± 2.7
Fraction of inspired oxygen	0.4 ± 0.17
Peripheral oxygen saturation D0, %	96 ± 2
Duration of mechanical ventilation, days	3 (2.25–7)
Hematologic and inflammatory data	
Leukocytes D0, cells/mm^3^	18,974 ± 9790
Leukocytes D7, cells/mm^3^	13,270 ± 6262
Hemoglobin D0, g/dl	10.4 ± 1.6
Hemoglobin D7, g/dl	9.7 ± 1.7
Platelets D0, 10^3^ cells/mm^3^	185 ± 134
Platelets D7, 10^3^ cells/mm^3^	261 ± 114
CRP D0 (*n* = 18), mg/dl	290 (212–327)
CRP D7 (*n* = 17), mg/dl	51 (30–125)
Procalcitonin D0 (*n* = 9), ng/ml	15.7 (2–22.4)
SAPS3 score	57.7 ± 6.2
SOFA score D0	8.4 ± 3.3
SOFA score D7	1.7 ± 3.8
ICU length of stay, days	5 (4–7.75)
Hospital length of stay, days	13.5 (8–22.5)
Hospital mortality, *n* (%)	8 (35)

*Abbreviations: PEEP* Positive end-expiratory pressure, *CRP* C-reactive protein, *SAPS3* Simplified Acute Physiology Score III, *SOFA* Sequential Organ Failure Assessment, *ICU* Intensive care unit

Data are expressed as number (%), mean ± SD, or median (25th–75th percentile)

### Characterization of exosomes

The EV isolated from plasma were evaluated by immunoblotting. We observed the expression and quantification of flotillin-1, a membrane-associated protein involved in endocytosis and an exosomal marker [25] (Additional file 1: Figure S1a and b). The RNA content was enriched mainly by miRNAs and RNAs in the size range of 19–400 nucleotides (Additional file 1: Figure S1c). Further analysis with NanoSight in three patients with paired samples collected at both time points demonstrated a size range predominantly from 90 to 150 nm (Additional file 1: Figure S1d), which is mostly consistent with exosomes.

The analysis of flow cytometry experiments demonstrated that a significant portion of vesicles was positive for CD9 and CD41 in both patients with sepsis and healthy volunteers, suggesting an exosomal and platelet origin for these vesicles (Additional file 1: Figure S2). Taken together, our results strongly suggest that the major component of our vesicle pool was composed of platelet-derived exosomes. Plasmatic concentrations of cytokines were below the detection limit of the method for the majority of the patients, except for IL-6 measurement in three patients (data not shown).

### Sepsis significantly alters the circulating exosomal miRNA profile

**Fig. 1:**
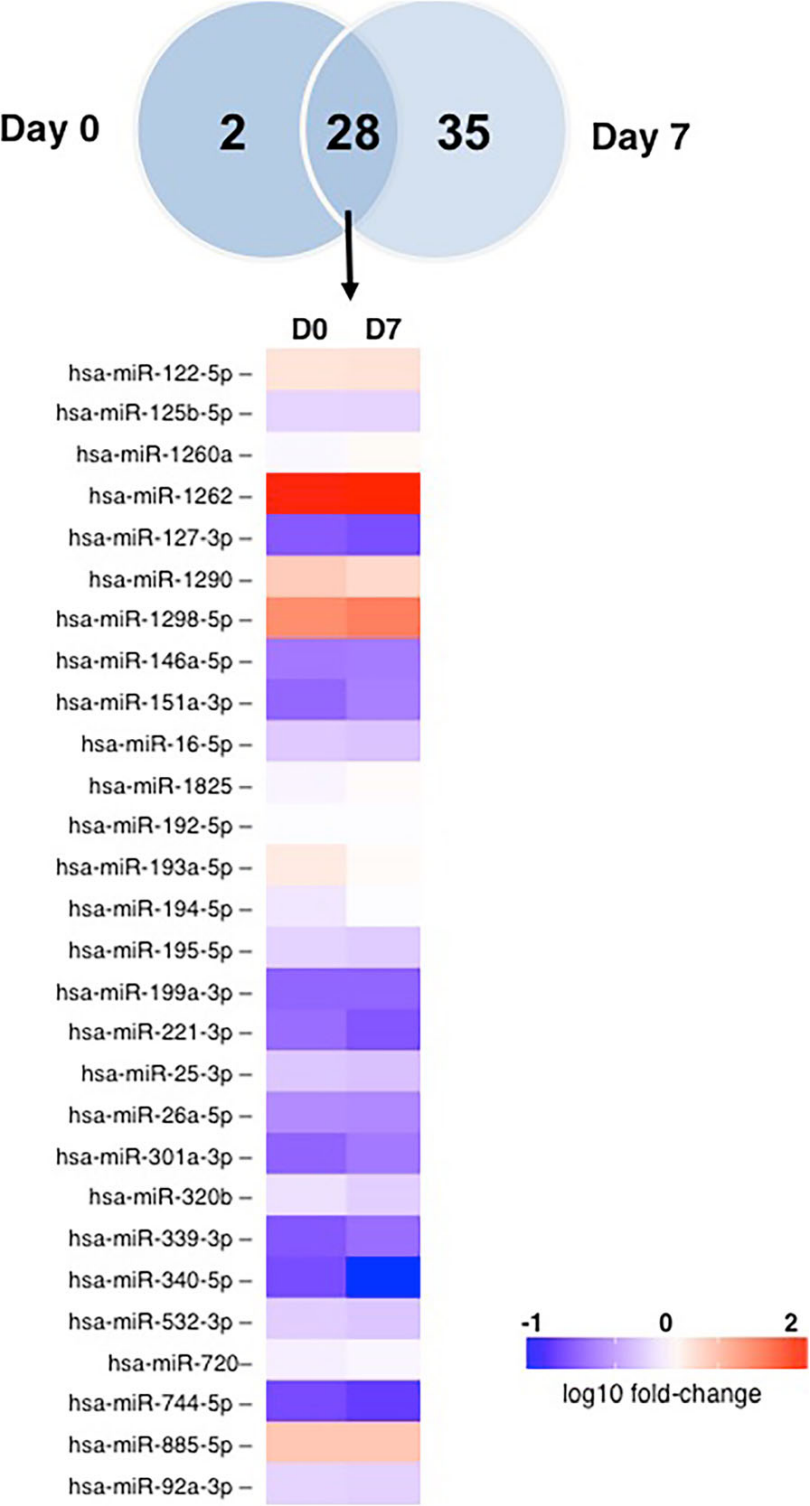
Exosomal RNA profile during sepsis. Venn diagram shows the number of microRNAs (miRs) from patients with sepsis at ICU admission (D0) and after 7 days (D7) as compared with those of healthy control subjects. The heat map illustrates the fold change profile of the 28 microRNAs differentially expressed at both time points

**Fig. 2:**
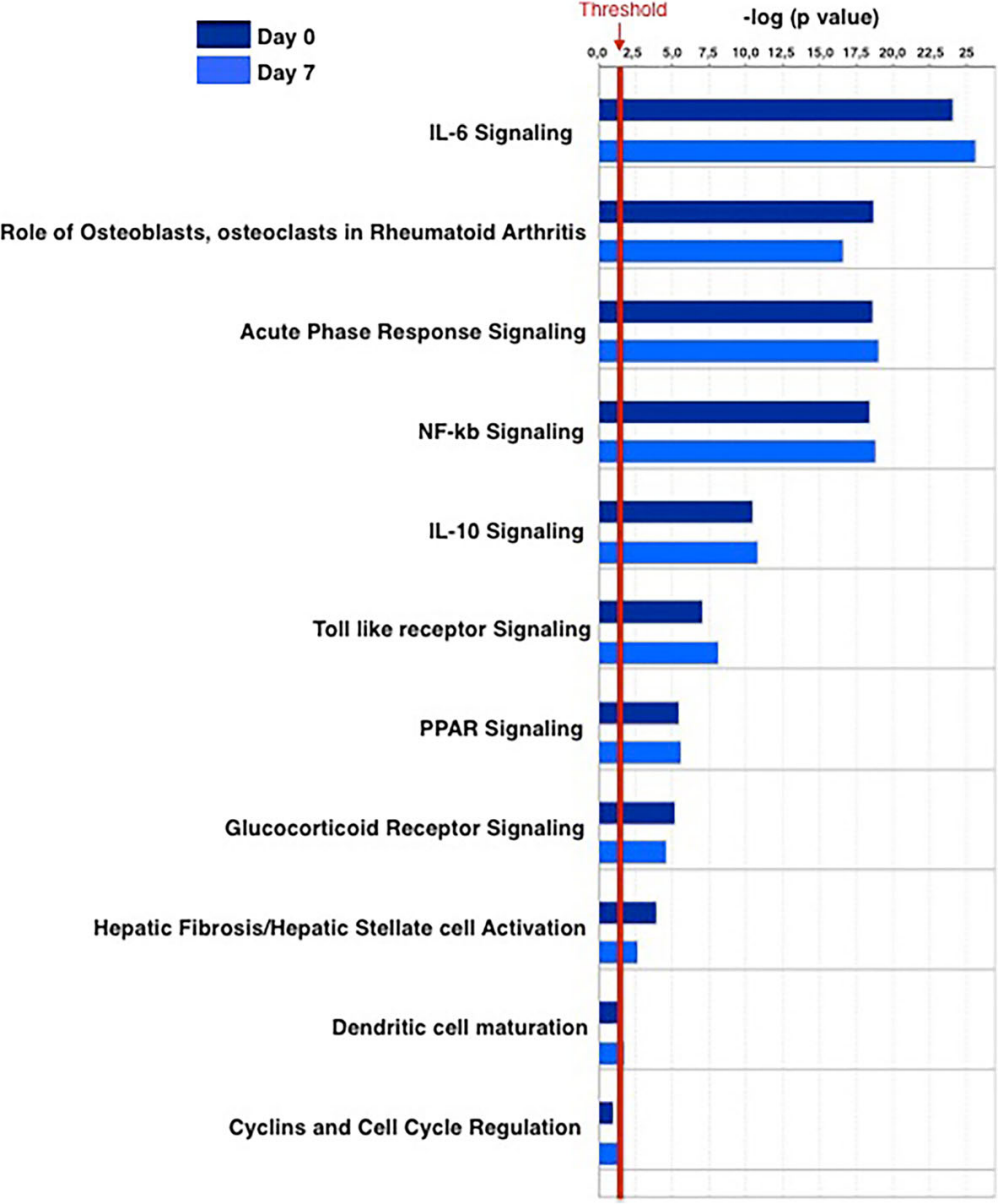
Biological pathways potentially influenced by differentially expressed microRNAs carried by exosomes of patients with septic shock at intensive care unit admission and 7 days later compared with those of healthy individuals. *NF-kB* Nuclear factor-κB, *PPAR* Peroxisome proliferator-activated receptor, *IL* Interleukin

To test the hypothesis that sepsis alters the expression of exosomal miRNAs, we first performed miRNA profiling analysis in patients with sepsis at enrollment and after 7 days and compared the results with those in healthy individuals (Additional file 1: Table S1). Thirty miRNAs were differentially expressed at enrollment as compared with controls. After 7 days, 65 miRNAs were differentially expressed between the sepsis and control samples. Twenty-eight miRNAs were commonly expressed at both time points (at enrollment and 7 days later). Figure 1 demonstrates that theses 28 miRNAs expressed at both time points maintained the same expression kinetics during the disease course (18 upregulated and 10 downregulated). Hierarchical clustering was performed for all samples considering the 65 differentially expressed miRNAs in sepsis. On the basis of miRNA expression values, samples from each group (septic and control) partially clustered together (Additional file 1: Figure S3). Regarding patients who had two samples collected during intensive care treatment, no miRNAs were differentially expressed between D0 and D7 (Wilcoxon paired-samples test). To gain insight into the biological significance of the global or system-level impacts of the differentially expressed miRNAs in exosomes in sepsis, IPA software was used to identify potential mRNA targets of the differentially expressed miRNAs. Considering only previously demonstrated targets of the miRNAs described here, 473 and 817 targets were identified at D0 and D7, respectively. The canonical pathways modulated by exosomal miRNAs at these two time points are mostly related to inflammatory and immune responses (Fig. 2). A schematic figure of IL-6 signaling canonical pathways showing the miRNAs differentially expressed in our study, as well as their targets, is shown in Additional file 1: Figure S4.

### Differential miRNA expression in patients with sepsis according to outcomes

**Fig. 3:**
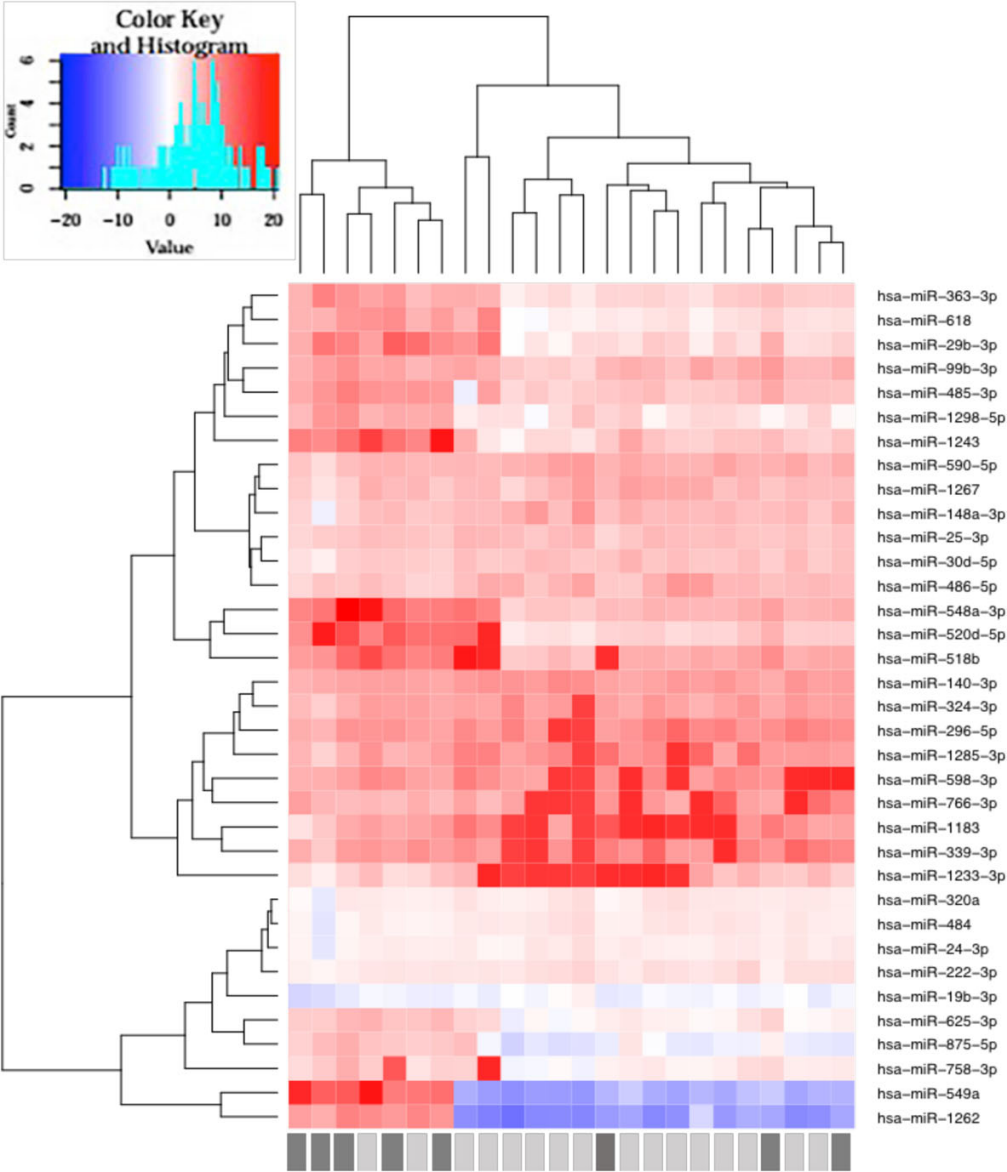
Patterns of microRNA (miR) expression from exosomes of patients with septic shock at enrollment (D0), comparing hospital survivors (*gray bars*) and nonsurvivors (*black bars*). The figure shows the analysis by hierarchical clustering of samples considering the change in cycle threshold values after filtering and normalization of data

To identify circulating miRNAs that could be related to clinical outcome, we further investigated the samples collected from patients with sepsis at ICU admission and analyzed their miRNA expression according to hospital outcomes. The unsupervised hierarchical clustering analysis (Fig. 3) showed a partial separation based on the expression of 35 miRNAs (Additional file 1: Table S2) of the patients who survived versus those who died of sepsis.

**Fig. 4:**
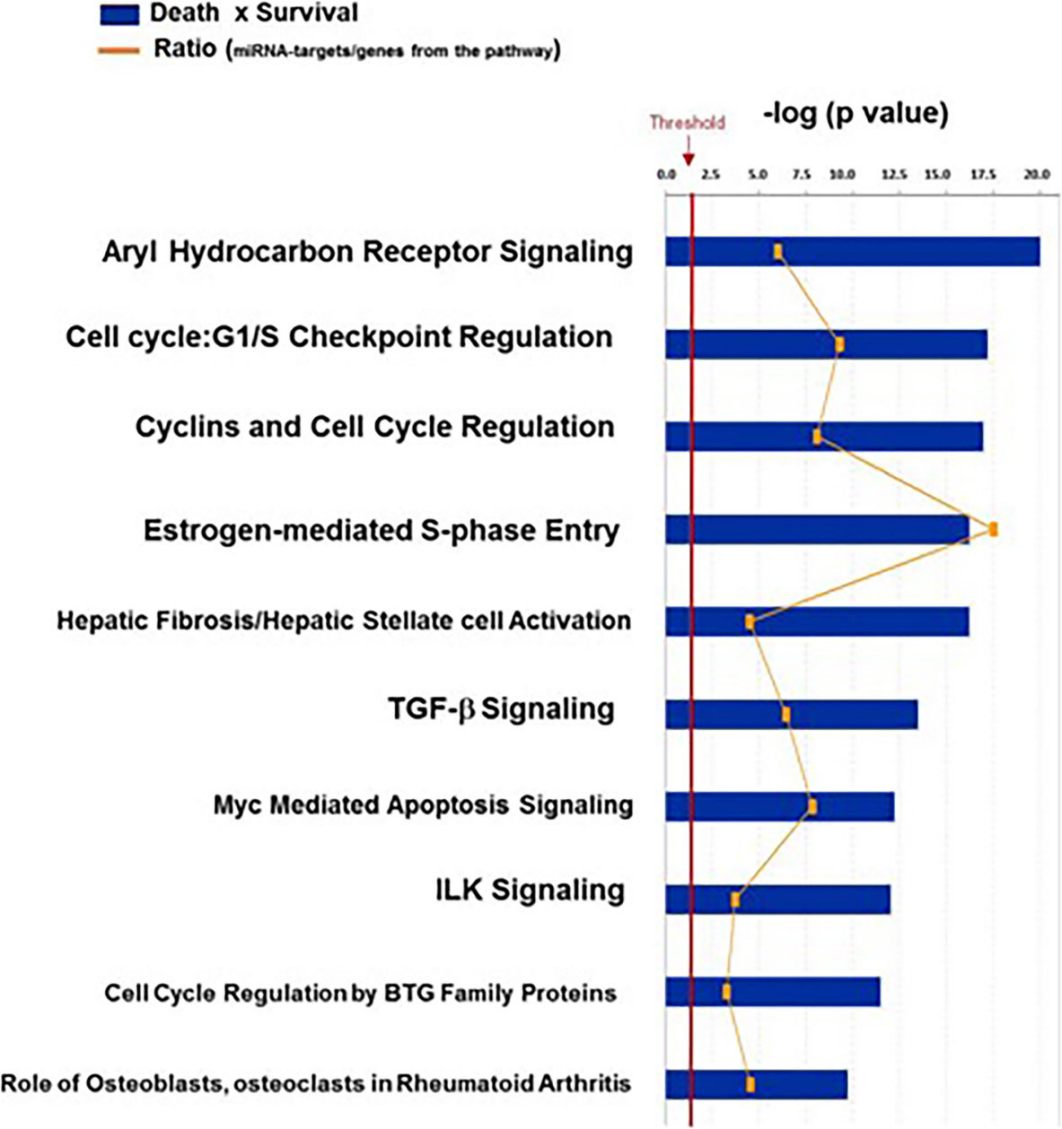
Enriched canonical pathways regulated by expressed microRNAs (miRNAs) in exosomes of patients with sepsis at enrollment according to hospital outcome. Ratios refer to the number of genes in the miRNA target list of a specific time point over the total number of genes in the respective canonical pathway. *TGF-β* Transforming growth factor-β, *ILK* Integrin-linked kinase, *BTG* B cell translocation gene

Enrichment pathway analysis was also performed to identify targets for these 35 differentially expressed septic exosomal miRNAs. We identified 275 specific experimentally validated cellular targets of these miRNAs. Differently from the comparison to healthy volunteers, the canonical pathways possibly influenced by these miRNAs were predominantly related to processes involving cell cycle regulation (Fig. 4). A schematic figure of cell cycle regulation and miRNAs that modulate it is provided in Additional file 1: Figure S5.

### Analysis of mRNAs related to inflammatory response

To evaluate whether mRNAs related to immune response were present in exosomes, we compared the expression of 90 immune/inflammation-related genes by qPCR using the RNA isolated from patients with sepsis at enrollment and after 7 days and from healthy individuals. We could not detect significant amplification of many of the immune/inflammation-related genes in the control group, so evaluation of patients with sepsis versus control subjects was not possible. However, considering a paired comparison of patients with sepsis between enrollment and 7-day samples, we observed that chemokine (C-C motif) ligand 5/regulated on activation, normal T cell expressed and secreted expression was 4.5-fold higher at the seventh day (*p* = 0.009; Wilcoxon paired-samples test).

### Exosomes from patients with sepsis contain mRNA related to redox signaling

**Fig. 5:**
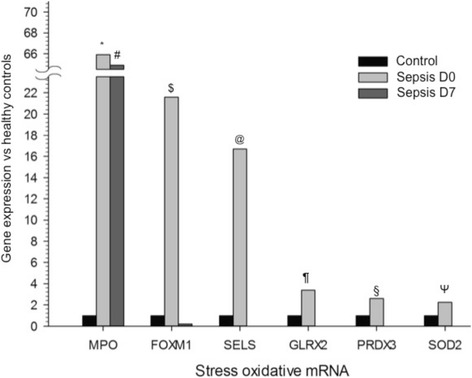
Gene expression of messenger RNA (mRNA) related to oxidative stress in the comparison between patients with sepsis (enrollment and 7 days later) versus healthy control subjects. * Unadjusted *p* value < 0.001; adjusted *p* value = 0.003. ^#^ Unadjusted *p* value < 0.001; adjusted *p* value = 0.008. ^$^ Unadjusted *p* value = 0.001; adjusted *p* value = 0.03. ^@^ Unadjusted *p* value = 0.004; adjusted *p* value = 0.04. ^¶^ Unadjusted *p* value = 0.005; adjusted *p* value = 0.04. ^§^ Unadjusted *p* value < 0.001; adjusted *p* value = 0.01. ^Ψ^ Unadjusted *p* value = 0.007; adjusted *p* value = 0.05. Unadjusted *p* value refers to Wilcoxon test. Adjusted *p* value refers to Benjamini-Hochberg correction for multiple comparisons. *n* = 21 for sepsis D0; *n* = 16 for sepsis D7; and *n* = 10 for controls. *MPO* Myeloperoxidase, *FOXM1* Forkhead box protein M1, *SELS* Selenoprotein S, *GLRX2* Glutaredoxin 2, *PRDX3* Peroxiredoxin 3, *SOD2* Superoxide dismutase 2

As shown in Fig. 5, we also investigated the expression of 84 mRNAs related to antioxidant defenses and oxidative stress in exosomes from patients with sepsis and healthy control subjects. As compared with healthy volunteers, patients with sepsis at enrollment had an increased expression of mRNAs of myeloperoxidase (MPO) (66-fold), the antioxidant enzyme peroxiredoxin 3 (2.6-fold), the mitochondrial enzyme superoxide dismutase 2 (2.2-fold), and the following oxidative stress response genes: Forkhead box protein M1 (FOXM1) (21-fold), selenoprotein S (16-fold), and glutaredoxin 2 (3.4-fold). After 7 days (D7), only MPO mRNA expression remained upregulated as compared with the control group (65-fold). There was no differentially expressed mRNA in the comparison of patients with sepsis between enrollment and 7 days later.

## Discussion

Our study comprehensively assessed the profile of miRNAs and mRNAs of exosomes from patients with septic shock in two stages of treatment. We demonstrated a differential regulation of miRNAs during sepsis, and the analysis of their targets indicates that they may potentially influence the immune system. Interestingly, the same pathways are still modified after 7 days of ICU admission, when most patients were already in a clinical recovery phase, as suggested by their reduced Sequential Organ Failure Assessment score. Our results suggest a subclinical and prolonged inflammatory response in sepsis, as previously reported in severe community-acquired pneumonia [26].

Earlier data demonstrated the role of miRNAs in the inflammatory/immune response in sepsis [27] and trauma. A study in which researchers analyzed the miRNA profiles of ten patients with polytrauma identified some miRNAs that were also differentially expressed in our patients (hsa-let-7b-5p, hsa-miR-151a-3p, hsa-miR-16-5p, hsa-miR-186-5p, hsa-miR-221-3p, hsa-miR-25-3p, hsa-miR-28-5p, hsa-miR-340-3p, and hsa-miR-618) [28]. This could indicate a similar response of the body to a serious infectious or inflammatory injury.

Very few previous studies demonstrated the presence of miRNAs in exosomes during sepsis. In a mouse model, miRNA-125b was identified in endothelial progenitor cell-derived exosomes, and sepsis induced a downregulation of this miRNA [19]. In our study, miRNA-125b showed greater expression in patients with sepsis with persistent expression after 7 days. In another study, some miRNAs from endothelial progenitor cell-derived exosomes (miR-15a, miR-27a, and miR-34a) had differential expression in plasma of patients with sepsis [18]. In our study, the expression of exosomal miR-27a was six times higher among sepsis survivors. These miRNAs modulate the inflammatory response and the cell cycle [18], a finding similar to that derived from our analysis.

Whereas the comparison between sepsis and controls identified miRNAs predominantly related to inflammation, the evaluation regarding outcomes identified miRNAs mostly related to cell cycle modulation. Cell cycle modulation was described in the context of sepsis and acute kidney injury [29–31] and correlated with miRNA regulation [29, 32]. In this scenario, miRNA regulation of target genes may lead the reentry into the proliferative cell cycle and differentiation, such as in fibrotic repair responses following tissue injury [33], or, more likely, miRNAs may regulate cell cycle arrest to prevent differentiation of damaged cells under hostile conditions [34, 35]. Whether it is conceivable that the miRNAs described here may affect both mechanisms, it is not possible without functional studies or on the basis of our results to identify which pathways are more influenced by these miRNAs.

The evaluation of mRNA related to oxidative stress demonstrated a significant and persistent increase in MPO mRNA. One major pathway regulated by MPO is the degradation of melatonin [36], which is reduced during sepsis, with MPO playing a role in this process [37, 38]. Another mRNA identified is FOXM1, a transcription factor related to cell cycle progression and activated by oxidative stress [39]. Overexpression of FOXM1 in septic mice protects against lung injury and increases survival [40, 41]. In our study, the increase in FOXM1 mRNA could also indicate a mechanism related to cell cycle regulation.

There is a paucity of information on how exosomal miRNAs interact with their targets to produce their effects. Most authors agree that exosomes are efficient carriers of proteins and genetic material to neighboring or more distant cells. However, the mechanisms of exosome–recipient cell interaction are unclear. The interaction usually starts with exosome internalization by various proposed pathways, including clathrin-mediated endocytosis, phagocytosis, or macropinocytosis [42]. Once in the recipient cell, exosomal miRNA and mRNA are usually functional and can interact with their targets to synthesize new proteins or modulate gene expression [13]. This has been demonstrated mostly in laboratory cell lines but not in sepsis, so any assumption regarding the interplay between exosomal genetic content and target cells during sepsis would be speculative.

Our study has limitations worth mentioning. First, this is an exploratory study with a small number of patients included, so our findings should be considered hypothesis-generating. Also, several comparisons were made, and as such, our analysis was corrected with a multiple correction-specific test (Benjamini-Hochberg) to minimize false-positive results. We included only patients in the first episode of sepsis to avoid those with chronic inflammation or immunosuppression that could influence the results. This criterion may limit the generalizability of our findings. Because we did not quantify the number of exosomes in the plasma of patients and healthy volunteers, the effect of sepsis on the exosomal counts was not estimated, and we could not normalize exosomal miRNA and mRNA levels to the amount of plasma exosomes. Plasmatic concentrations of cytokines were below the detection limit of the method in our patients, probably owing to the long-term storage of these samples, so we could not correlate the results of inflammatory cytokines with the exosomal genetic content. Also, we assessed only mRNA related to inflammatory response and oxidative stress, because these two important mechanisms were previously demonstrated to mediate the effects of exosomes in sepsis [11, 12]. With this approach, it was not possible to evaluate other pathophysiological targets of exosomal mRNA. In order to compare the effects of infectious and noninfectious injury, we did not include a control group composed of noninfected ICU patients. In addition, we did not evaluate the function of these miRNAs in sepsis, so the pathways that they influence were not demonstrated in this study.

## Conclusions

Exosomes from patients with sepsis convey genetic material that may be related to key pathways in the pathogenesis of sepsis, including inflammatory response, oxidative stress, and cell cycle regulation. Further functional studies are required to clarify the exact contribution of these vesicles to the exchange of genetic material and intercellular communication during sepsis.

## Additional file

Additional file 1: Supplementary methods and results. (PDF 4801 kb)

## Abbreviations

BTG: B cell translocation gene
cDNA: Complementary DNA
CRP: C-reactive protein
*C*_t_: Cycle threshold
2^−ΔΔ*C*t^: Comparative cycle threshold method
EV: Extracellular vesicles
FOXM1: Forkhead box protein M1
GLRX2: Glutaredoxin 2
ICU: Intensive care unit
IL: Interleukin
ILK: Integrin-linked kinase
IPA: Ingenuity Pathway Analysis
miRNA: miR, MicroRNA
MPO: Myeloperoxidase
mRNA: Messenger RNA
NF-κB: Nuclear factor-κB
PEEP: Positive end-expiratory pressure
PPAR: Peroxisome proliferator-activated receptors
PRDX3: Peroxiredoxin 3
SAPS: Simplified Acute Physiology Score
SELS: Selenoprotein S
SOD2: Superoxide dismutase 2
SOFA: Sequential Organ Failure Assessment
TGF-β: Transforming growth factor-β
