# Supplementary material for: Exosomes from patients with septic shock convey miRNAs related to inflammation and cell cycle regulation: new signaling pathways in sepsis?
Source: Crit Care. 2018 Mar 15;22:68. doi: 10.1186/s13054-018-2003-3 (PMC5852953; doi:10.1186/s13054-018-2003-3)
Supplement: Supplementary file 1 — Supplementary methods and results. (PDF 4801 kb) [file 13054_2018_2003_MOESM1_ESM.pdf]

Exosomes from septic shock patients convey miRNAs related to inflammation and cell cycle regulation: new signaling pathways in sepsis?

Juliana Monte Real; Ludmila Rodrigues Pinto Ferreira; Gustavo Henrique Esteves; Fernanda Christtanini Koyama; Marcos Vinícius Salles Dias; João Evangelista Bezerra-Neto; Edécio Cunha Neto; Flavia Ribeiro Machado; Reinaldo Salomao; Luciano Cesar Pontes Azevedo.

Supplementary Digital Content

Material and Methods

Inclusion and Exclusion Criteria

Patients were recruited if diagnosed with less than 24 hours of septic shock (1). We excluded patients with hemoglobin <7.0 g/dL, with known platelet diseases or conditions causing thrombocytopenia other than sepsis, use of full heparin or any other medications that interfere with platelet function. Moribund or active cancer patients were also excluded. Disease severity was evaluated by SAPS 3 score (2) and SOFA (sequential organ failure assessment) (3).

Blood Sample Collection, Processing and Plasmatic Cytokines Measurement

Blood sample (30 mL) was collected on EDTA tubes and maintained at 4°C until being processed (maximum of 2 hours). Protease inhibitors (3 mM phenylmethylsulfonyl fluoride, 1 µg/ml aprotinin and 1 µg/ml pepstatin) were added and plasma was stored frozen at -80°C. Plasma was filtered through a 0.22 µm filter and microvesicles were isolated by ultracentrifugation at 120,000g for 2h30 at 4º C (SW 28 Ti Rotor, Optima L-90K Ultracentrifugue, Beckman Coulter, Fullerton, USA) (4). Supernatants were discarded and the pellets were washed twice with PBS. MicroRNA and total RNA were extracted using the miRNeasy Mini Kit (Qiagen, Hilden, Germany), according to the manufacturer's protocol. RNA quantity and quality were assessed by spectrophotometry (NanoVue, GE Healthcare, Munich, Germany) and microchip electrophoresis (Bioanalyzer 2100, Agilent Technologies, Palo Alto, United States) respectively.

In a subgroup of seven patients, we measured plasmatic concentrations of IL-1b, IL-6, IL-8, IL-10, IL-13, TNF-alpha and TGF-beta by Enzyme-Linked Immunosorbent Assay according to the manufacturers instructions (R&D Systems, Minnesota, USA).

Western Blotting

Protein was extracted from 1 ml of ultracentrifugated plasma (120,000g for 2h30) using DE buffer (20 mM Tris-HCL, 1 mM EDTA, 1 mM EGTA, 12 mM 2-mercaptoethanol, 10% glycerol, 1% Triton-X 100) (5) with protease inhibitor mix (GE Healthcare, Uppsala, Sweden). Total protein was quantified by Bradford and 5 µg of protein per sample were separated by 12% SDS-PAGE. Proteins were transferred to nitrocellulose and blotted with the polyclonal antibody against Flotillin-1 (ABcam

ab41927, Cambridge, UK). Coomassie blue staining of the membrane was used as a loading control for the protein extracts (6). The quantification of Flotillin-1 was performed by densitometry analysis using the ImageJ software. Each band was reduced from the gel background.

Exosomes' size distribution profiles and concentration measurements

Circulating exosomes from six patients with samples collected at enrollment and seven days later were quantified and sized. Exosomes were isolated from 100µL of plasma as described above. Three videos of 60 seconds were recorded and analyzed using NanoSight LM10 and NTA software (NanoSight Ltd, Amesbury, UK).

Flow Cytometry Analysis

The pellet concentrated with exosomes from eight septic patients at each time point and eight healthy controls isolated as described was thawed in room temperature and 10 µL of each sample was used in the staining protocol. We used 1-2.5 µL of human monoclonal antibodies (mAb) anti-CD9 (CBL 162 – Cymbus Biotechnology, Southampton, United Kingdom) and CD41-APC (Beckman Coulter, Indianapolis, USA) for 20 minutes at room temperature. For CD9 experiments, 1 µL of FITC conjugated secondary antibody (Abcam#6669-1) was added and incubated for another 20 minutes. We also prepared single staining sample as compensation controls as well as samples without exosomes and exosomes samples incubated only with secondary antibodies.

All acquisitions were done using the CytoFLEX flow cytometer (Beckman Coulter). We used the Violet Side Scatter (VSSC) and fluorescent polystyrene beads (megamix FSC & SSC Plus, Biocytex, Marseille, France) with known sizes (100, 160, 200, 240, 300, 500 and 900 nm) to better identify vesicles lower than 1 µm. Because

coincidence events compromise the quality of data when exosomes are measured by flow cytometry, we diluted the sample at 1:200 and acquired the data in a maximum flow rate of 1200 events per second. The analysis was performed using the software Cytexper 2.1 (Beckman Coulter). We used a sequence of negative and single staining controls in order to ensure the confidentiality of our results. The CD9-marked exosomes were identified through the CD9 expression vs VSSC dot plot, excluding the blank events. The CD41-derived exosomes were gated on $CD9^+$ exosomes and identified through the CD41 expression Vs VSSC dot plot.

Analysis of microRNA and inflammatory gene expression

Thirty nanograms of total RNA was used to reverse transcribed the miRNAs using specific stem-loop primers. The preamplification reaction products were analyzed by qPCR using the TaqMan Low Density Array (TLDA), a micro fluidic card that simultaneously detects 754 mature miRNAs (TaqMan MicroRNA Array v3.0, Life Technologies, Grand Island, EUA). The immune study was performed in 21 septic patients and 12 controls. Reverse transcription was performed from 100ng of RNA following the instructions of the “High Capacity RNA-to-cDNA Kit" (Life Technologies). The cDNA was distributed in TLDA cards designed to evaluate 90 genes implied in immune response (TaqMan Low Density Array Immune Profiling, Life Technologies).

Real-time PCR reactions were performed on the Applied Biosystems 7900HT thermocycler using SDS 2.4 software. All data collected were analyzed in the ExpressionSuite 1.0.3 software (Life Technologies), where baseline and threshold was automatically set. Each gene was checked individually and threshold was adjusted to exponential scale as needed.

Analysis of gene expression associated with Oxidative Stress

The PCR array platform used in these experiments comprises 84 genes associated with Oxidative Stress and Antioxidant Defense (PAHS065-A, SABiosciences/Qiagen, Valencia, USA). Twenty-one patients and ten healthy donors were evaluated. Following the manufacturer's instructions, 100ng of RNA were used for the reverse transcription followed by pre-amplification of cDNA using specific primers. In order to avoid variation, PCR plates were prepared in high precision robotic pipetting (QI Agility, Qiagen). Quantitative real time PCR was done in the 7300HT thermocycler (Life Technologies). The data were processed considering the same baseline (3-15) and threshold (0.2) for each gene using the SDS 1.3 software.

qPCR Data Analysis

All Cycle Threshold (Ct) values were analyzed in Statminer v5 (Integromics, Granada, Spain). According to the manufacturer's instructions, Ct values greater than or equal to 32 were excluded for miRNAs and immune studies. For oxidative stress studies, the cutoff value was established at 35 according to the manufacturer's instructions. For the miRNA analysis, the geNorm method was used to identify the best reference controls and the median of miR17, miR20a and miR106a was used for the data normalization. The expression levels of immune and stress oxidative genes were normalized to 18S rRNA (7) and beta-actin, respectively. We calculated the relative gene expression data by the 2^DDCt method (8).

Supplementary Results

FIGURES

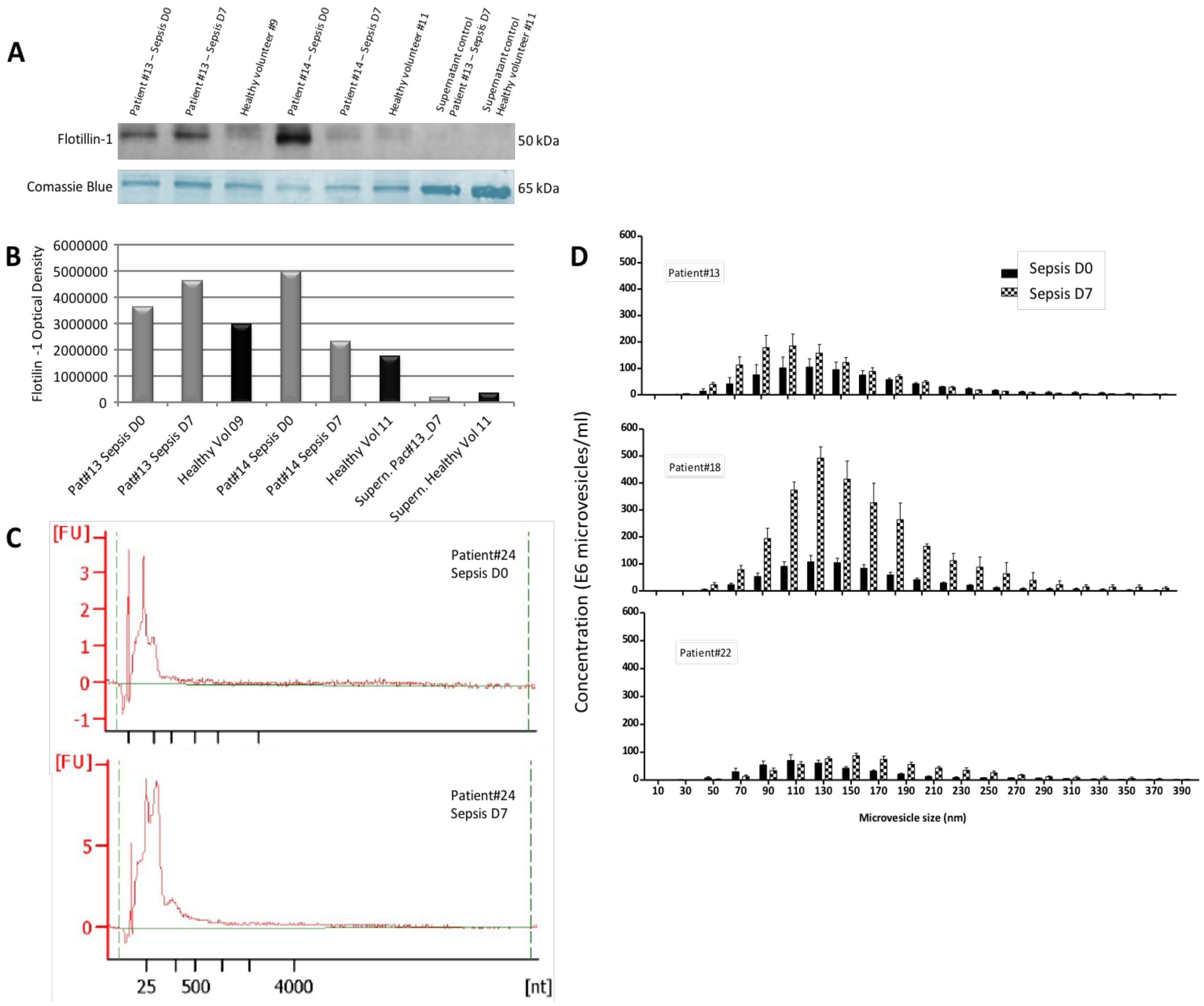

Supplementary Fig S1. Characterization of exosomes in plasma of septic shock patients and healthy controls. **Panel A.** Western blot analysis of Flotillin-1 exosomes isolated by ultracentrifugation. Lanes contain exosomes from septic patients collected at ICU admission and 7 days after and from healthy volunteers. No flotillin was detected in the supernatant after ultracentrifugation. **Panel B.** Protein quantification of Flotilin-1 from the Western blot results with ImageJ software. **Panel C.** Total RNA profile of exosomes from a septic shock patient collected at ICU admission and D7 and determined by electrophoresis using RNA Pico Chip in

Bioanalyser. **Panel D.** Exosomes' size distribution profile in sepsis. The graphs represent the plasma exosomes' concentration in three patients at admission (black bars) and seven days later (black/white bars). Exosomes were isolated from plasma by ultracentrifugation and evaluated in Nanosight. Representative experiments.

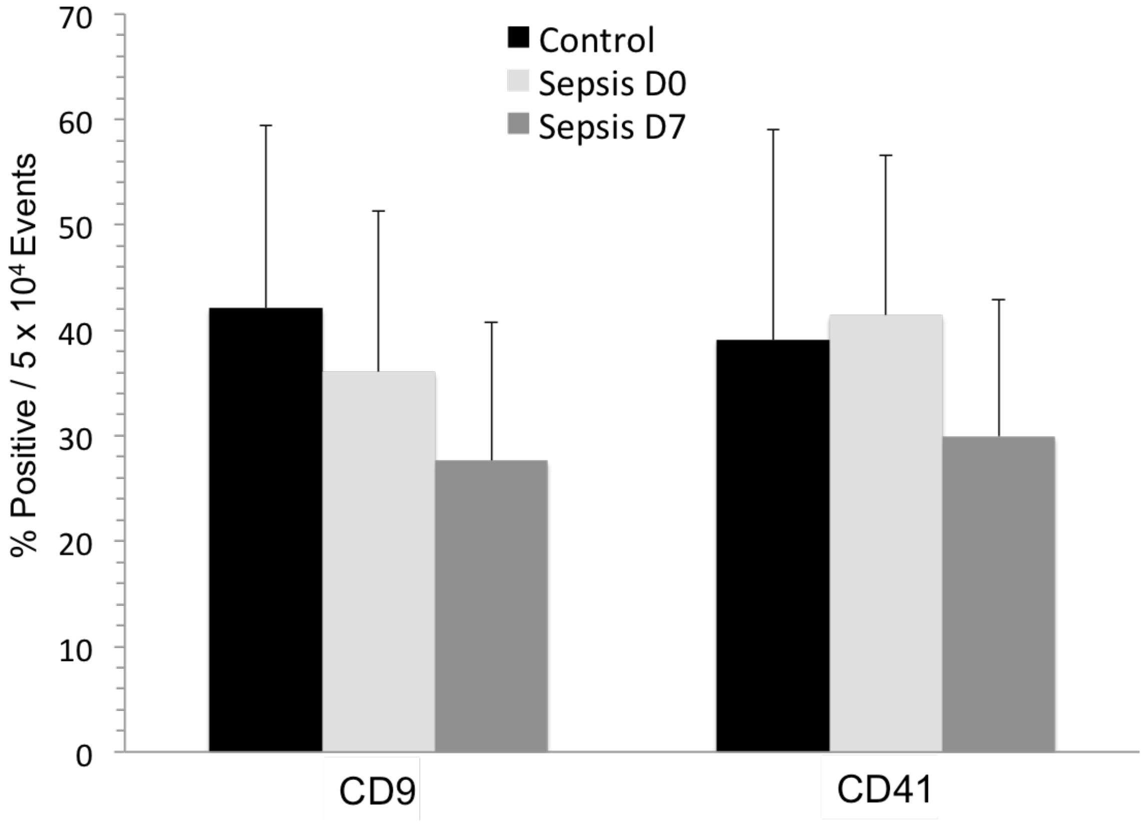

Supplementary Fig S2. Characterization of exosomes in plasma of septic shock patients and healthy controls by nano flow cytometry. The graph depicts the percentage of positive events of 50,000 vesicles. Data are mean ± SD of eight experiments for septic patients at each time point and eight controls. Exosomes from septic patients and healthy controls were incubated with CD9 (exosome marker) and CD41 (platelet marker).

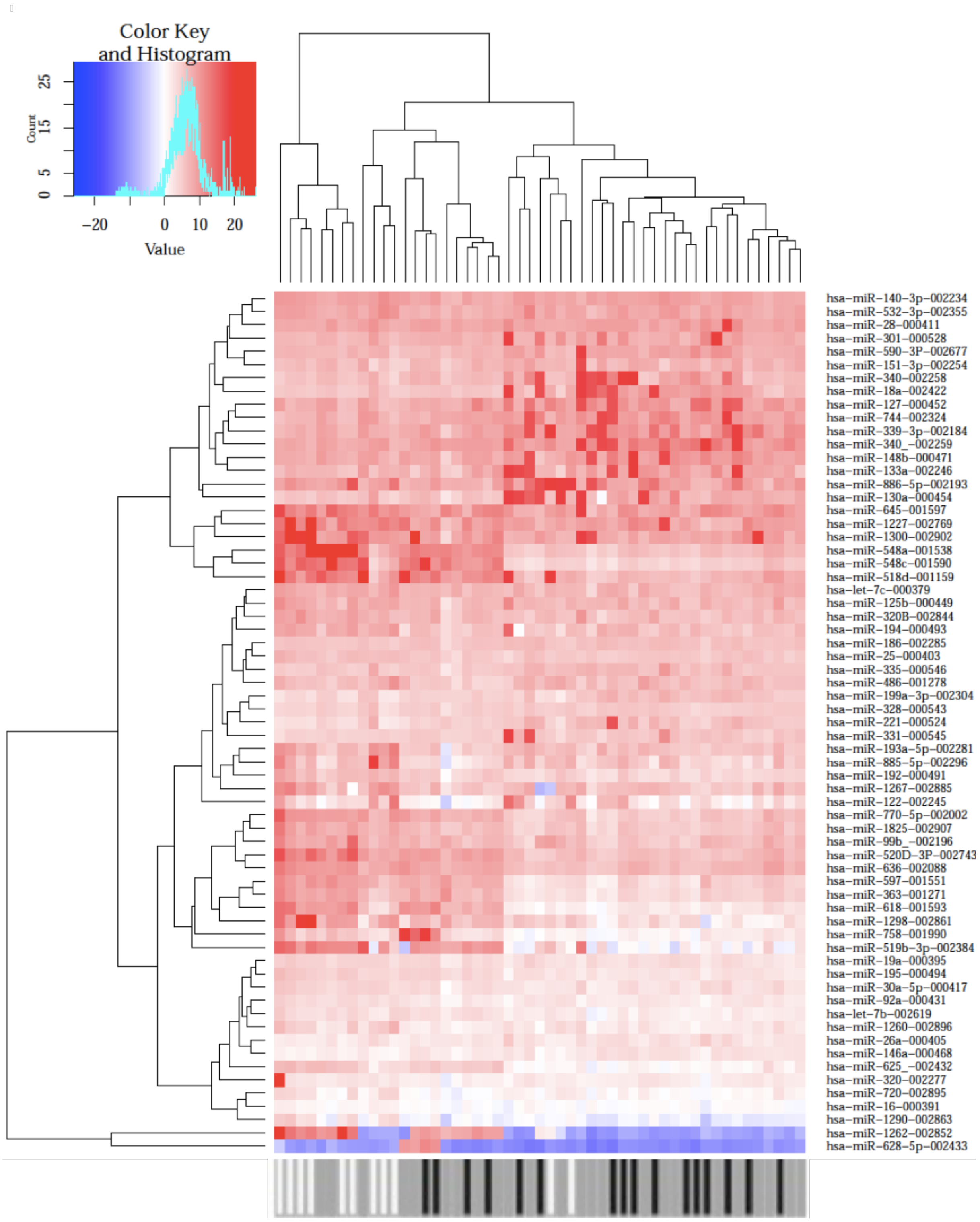

Supplementary Fig S3. Heatmap of the normalized DCt values profile of 65 exosomal microRNAs of septic shock patients at admission (D0 - grey bars) and seven days later (D7 - black bars), and healthy controls (white bars).

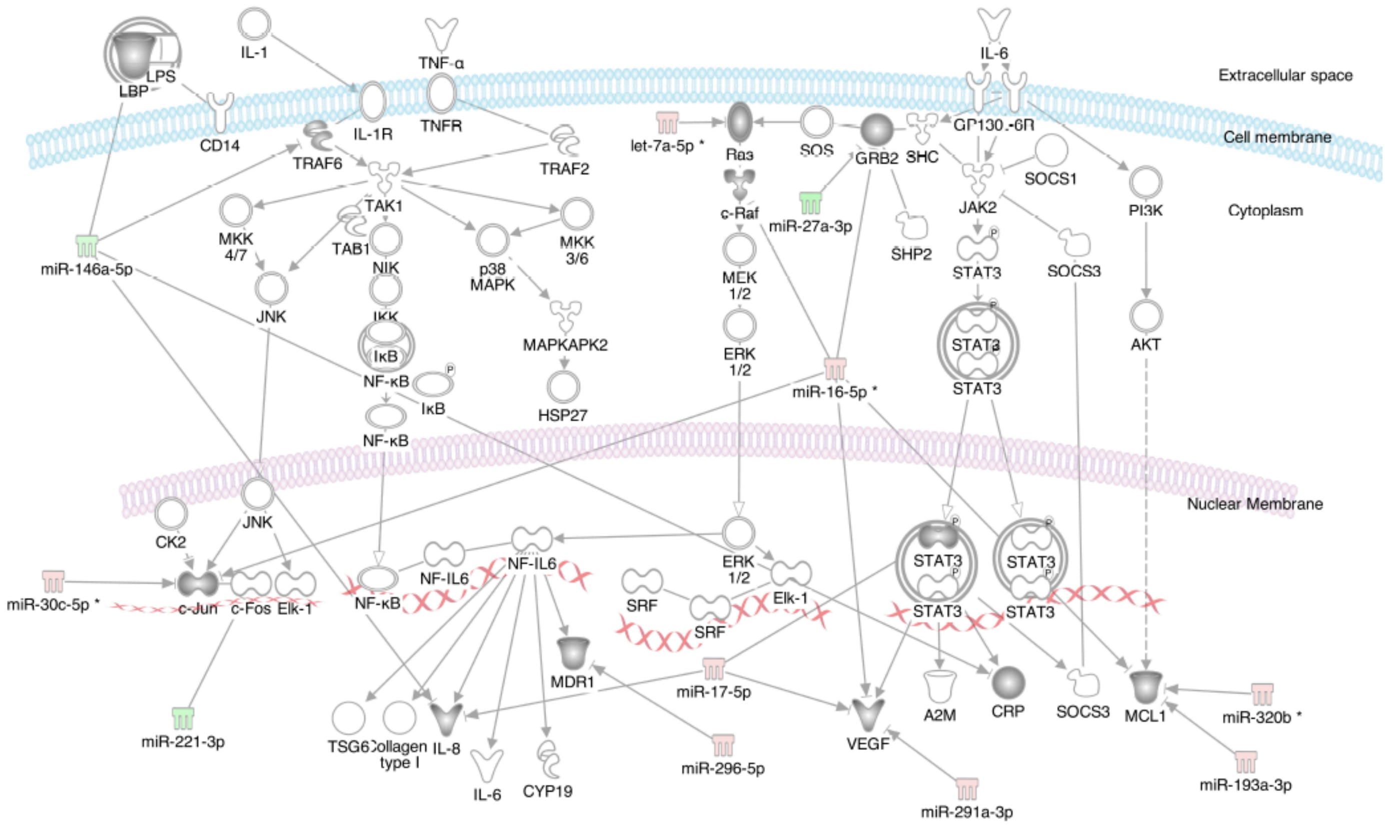

Supplementary Fig S4. Targets of exosomal miRNAs expressed in sepsis involved in the IL-6 signaling canonical pathway. Green colors represent miRNAs underexpressed and red colors represent miRNAs overexpressed.

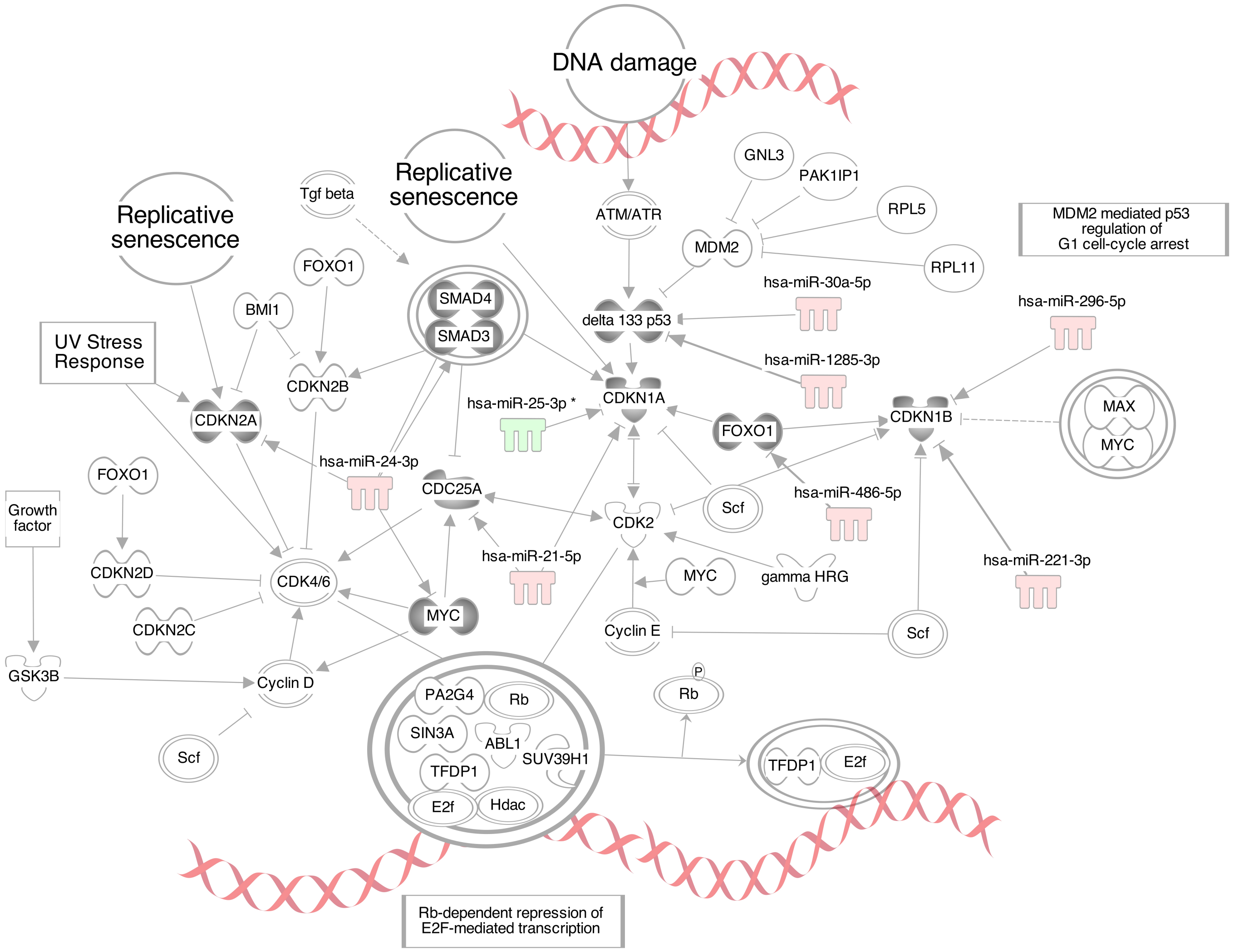

Supplementary Fig S5. Canonical pathways related to cell cycle regulation modulated by miRNA contained in exosomes from septic patients differentially expressed according to hospital outcome. Green colors represent miRNAs underexpressed and red colors represent miRNAs overexpressed.

TABLES

Supplementary Table S1. MicroRNA expression profile of septic exosomes compared to healthy controls.

mirBase ID	Sepsis D0 vs Control			Sepsis D7 vs Control		
	Fold Change	Unadjusted P value	Adjusted P value	Fold Change	Unadjusted P value	Adjusted P value
hsa-let-7b-5p	—	—	—	2.3	0.017	0.034
hsa-let-7c-5p	—	—	—	2.5	0.020	0.038
hsa-miR-122-5p	13.6	0.002	0.042	14.0	0.011	0.023
hsa-miR-1227-3p	—	0.005	—	15.1	0.009	0.020
hsa-miR-125b-5p	2.8	0.003	0.046	2.7	0.026	0.048
hsa-miR-1260a	5.8	<0.001	0.001	7.5	0.039	0.002
hsa-miR-1262	825.9	0.007	0.050	980.4	<0.001	0.030
hsa-miR-1267	—	—	—	4.9	0.015	0.023
hsa-miR-127-3p	-5.2	0.003	0.046	-6.6	0.011	0.009
hsa-miR-1290	25.3	<0.001	<0.001	17.9	0.004	0.002
hsa-miR-1298-5p	118.4	<0.001	0.003	167.7	<0.001	0.002
hsa-miR-1300	—	—	—	23.1	<0.001	0.034
hsa-miR-130a-3p	—	—	—	-7.4	0.017	0.004
hsa-miR-133a-3p	—	—	—	-7.9	0.001	0.005
hsa-miR-140-3p	2.2	0.001	0.024	—	—	—
hsa-miR-146a-5p	-2.7	<0.001	0.001	-2.5	0.001	0.004
hsa-miR-148b-3p	—	—	—	0.2	0.006	0.012
hsa-miR-151a-3p	-3.8	0.001	0.026	-2.3	0.001	0.004
hsa-miR-16-5p	2.2	<0.001	0.009	1.9	0.001	0.004
hsa-miR-1825	5.4	0.001	0.028	7.3	<0.001	0.004
hsa-miR-186-5p	—	—	—	-1.5	0.002	0.004
hsa-miR-18a-5p	—	—	—	-12.3	0.002	0.005
hsa-miR-192-5p	6.4	<0.001	0.001	6.4	<0.001	0.003
hsa-miR-193a-5p	11.1	<0.001	0.001	7.4	<0.001	0.004
hsa-miR-194-5p	4.1	0.001	0.016	6.4	<0.001	0.004
hsa-miR-195-5p	2.7	0.002	0.042	2.3	0.020	0.038
hsa-miR-199a-3p	-4.1	<0.001	0.003	-4.1	<0.001	0.004
hsa-miR-19a-3p	—	—	—	1.6	0.013	0.026
hsa-miR-221-3p	-3.4	0.002	0.038	-5.6	0.002	0.004
hsa-miR-25-3p	2.1	0.001	0.020	1.8	0.003	0.008
hsa-miR-26a-5p	-1.8	0.004	0.050	-1.9	0.004	0.009
hsa-miR-28-5p	—	—	—	-2.6	0.004	0.009
hsa-miR-301a-3p	-4.2	<0.001	0.006	-2.6	0.001	0.004
hsa-miR-30a-5p	—	—	—	2.0	0.020	0.038
hsa-miR-320a	—	—	—	6.6	0.023	0.043
hsa-miR-320b	3.5	0.002	0.029	2.5	0.020	0.038
hsa-miR-328-3p	—	—	—	-1.5	0.026	0.048
hsa-miR-331-3p	—	—	—	-2.3	<0.001	0.004
hsa-miR-335-5p	—	—	—	-1.7	0.015	0.030
hsa-miR-339-3p	-5.4	0.001	0.026	-3.3	<0.001	0.004
hsa-miR-340-5p	-6.1	0.002	0.05	-21.9	0.002	0.005
hsa-miR-340-3p	-6.9	0.004	0.029	—	—	—

hsa-miR-363-3p	—	—	—	6.3	0.020	0.038
hsa-miR-486-5p	—	—	—	2.1	0.026	0.048
hsa-miR-518d-3p	—	—	—	14.9	0.023	0.043
hsa-miR-519b-3p	—	—	—	71.5	0.011	0.023
hsa-miR-520d-3p	—	—	—	16.1	0.009	0.020
hsa-miR-532-3p	2.4	0.001	0.020	2.0	0.011	0.023
hsa-miR-548a-3p	—	—	—	44.8	0.011	0.023
hsa-miR-548c-3p	—	—	—	13.8	0.023	0.043
hsa-miR-590-3p	—	—	—	-3.0	0.008	0.017
hsa-miR-597-5p	—	—	—	8.6	0.006	0.012
hsa-miR-618	—	—	—	14.3	0.017	0.034
hsa-miR-625-3p	—	—	—	6.2	0.004	0.009
hsa-miR-628-5p	—	—	—	-1.6	0.006	0.012
hsa-miR-636	—	—	—	5.1	0.004	0.009
hsa-miR-645	—	—	—	7.1	0.003	0.006
hsa-miR-720	4.8	<0.001	0.004	5.5	<0.001	0.004
hsa-miR-744-5p	-7.2	<0.001	0.001	-9.7	<0.001	0.004
hsa-miR-758-3p	—	—	—	3.1	0.023	0.043
hsa-miR-770-5p	—	—	—	12.3	0.001	0.004
hsa-miR-885-5p	28.7	<0.001	0.002	28.9	<0.001	0.004
hsa-miR-886-5p	—	—	—	7.6	0.017	0.034
hsa-miR-92a-3p	2.7	<0.001	0.003	2.6	<0.001	0.003
hsa-miR-99b-3p	—	—	—	7.5	0.001	0.004

Unadjusted P-value refers to Wilcoxon test. Adjusted P-value refers to Banjamini-Hochberg correction for multiple comparisons.

Supplementary Table S2. MicroRNAs differentially expressed in septic patients' exosomes according to outcome.

mirBase ID	Sepsis D0 survivors vs non-survivors		
	Fold Change	Unadjusted P-value	Adjusted P-value
hsa-miR-1183	33.9	0.016	0.034
hsa-miR-1233-3p	55.3	0.019	0.041
hsa-miR-1243	-53.6	0.005	0.034
hsa-miR-1262	-2,288.1	0.007	0.034
hsa-miR-1267	3.3	0.009	0.034
hsa-miR-1285-3p	8.1	0.016	0.034
hsa-miR-1298-5p	-9.3	0.016	0.034
hsa-miR-140-3p	2.2	0.002	0.034
hsa-miR-148a-3p	4.7	0.007	0.034
hsa-miR-19b-3p	2.4	0.009	0.034
hsa-miR-222-3p	2.1	0.009	0.034
hsa-miR-24-3p	1.9	0.024	0.048
hsa-miR-25-3p	2.2	0.001	0.034
hsa-miR-296-5p	6.1	0.009	0.034
hsa-miR-29b-3p	-26.5	0.016	0.034
hsa-miR-30d-5p	2.9	0.001	0.034
hsa-miR-320a	2.3	0.016	0.034
hsa-miR-324-3p	3.8	0.017	0.037
hsa-miR-339-3p	12.7	0.011	0.034
hsa-miR-363-3p	-9.6	0.011	0.034
hsa-miR-484	2.7	0.000	0.034
hsa-miR-485-3p	-8.8	0.013	0.034
hsa-miR-486-5p	3.6	0.002	0.034
hsa-miR-518b	-7.9	0.019	0.041
hsa-miR-520d-5p	-63.5	0.019	0.041
hsa-miR-548a-3p	-20.4	0.024	0.048
hsa-miR-549a	-11,616.4	0.003	0.034
hsa-miR-590-5p	2.5	0.019	0.041
hsa-miR-598-3p	28.9	0.003	0.034
hsa-miR-618	-10.7	0.024	0.048
hsa-miR-625-3p	-7.3	0.005	0.034
hsa-miR-758-3p	-7.9	0.019	0.041
hsa-miR-766-3p	34.6	0.013	0.034
hsa-miR-875-5p	-18.9	0.011	0.034
hsa-miR-99b-3p	-3.3	0.011	0.034

Unadjusted P-value refers to Wilcoxon test. Adjusted P-value refers to Benjamini-Hochberg correction for multiple comparisons.

Supplementary References

1. Bone RC, Balk RA, Cerra FB, Dellinger RP, Fein AM, Knaus WA, Schein RMH, Sibbald WJ. Definitions for sepsis and organ failure and guidelines for the use of innovative therapies in sepsis. *Chest* 1992; p. 1644–1655.

2. Moreno RP, Metnitz PGH, Almeida E, Jordan B, Bauer P, Campos RA, Iapichino G, Edbrooke D, Capuzzo M, Le Gall JR. SAPS 3 - From evaluation of the patient to evaluation of the intensive care unit. Part 2: Development of a prognostic model for hospital mortality at ICU admission. *Intensive Care Med* 2005;31:1345–1355.

3. Jones AE, Trzeciak S, Kline JA, The Sequential Organ Failure Assessment score for predicting outcome in patients with severe sepsis and evidence of hypoperfusion at the time of emergency department presentation. *Crit Care Med* 2009;37:1649–1654.

4. Azevedo LCP, Janiszewski M, Pontieri V, Pedro MDA, Bassi E, Tucci PJF, Laurindo FRM. Platelet-derived exosomes from septic shock patients induce myocardial dysfunction. *Crit Care* 2007;11:R120.

5. Wolf M, Sahyoun N. Protein kinase C and phosphatidylserine bind to M(r) 110,000/115,000 polypeptides enriched in cytoskeletal and postsynaptic density preparations. *J Biol Chem* 1986;261:13327–13332.

6. Welinder C, Ekblad L. Coomassie staining as loading control in Western blot analysis. *J Proteome Res* 2011;10:1416–1419.

7. Miranda KC, Bond DT, Levin JZ, Adiconis X, Sivachenko A, Russ C, Brown D, Nusbaum C, Russo LM. Massively parallel sequencing of human urinary exosome/microvesicle RNA reveals a predominance of non-coding RNA. *PLoS One* 2014; 9(5):e96094.

8. Livak KJ, Schmittgen TD. Analysis of relative gene expression data using real-time quantitative PCR and the 2(-Delta Delta C(T)) Method. *Methods* 2001;25:402–408.
